# The effects of maternal and post-weaning diet interaction on glucose metabolism and gut microbiota in male mice offspring

**DOI:** 10.1042/BSR20160103

**Published:** 2016-06-03

**Authors:** Jia Zheng, Xinhua Xiao, Qian Zhang, Miao Yu, Jianping Xu, Cuijuan Qi, Tong Wang

**Affiliations:** * Department of Endocrinology, Key Laboratory of Endocrinology, Ministry of Health, Peking Union Medical College Hospital, Diabetes Research Center of Chinese Academy of Medical Sciences and Peking Union Medical College, Beijing 100730, China

**Keywords:** glucose homoeostasis, gut microbiota, high-fat diet, maternal nutrition, offspring

## Abstract

Substantial studies demonstrated that maternal nutrition can significantly determine the susceptibility to developing some metabolic diseases in offspring. However, investigations into the later-life effects of these diets on gut microbiota in the offspring are limited. Our objective was to explore the effects of maternal and post-weaning diet interaction on offspring's gut microbiota and glucose metabolism in later life. The male offspring of dams fed on either a high-fat (HF) diet or control (C) diet and then weaned to either a HF or C diet, generating four groups: C–C, HF–C, C–HF and HF–HF (*n*=8 in each group). The C–C offspring had lower body weight than C–HF group at 16 weeks of age (*P*<0.01) and both C–HF and HF–HF offspring had higher body weight than C–C group at 24 and 32 weeks of age (*P*<0.001 respectively). The blood glucose (BG) levels of the male offspring from the C and HF dams weaned HF diet were significantly higher at 30 min, 60 min and 120 min (*P*<0.001) after intraperitoneal glucose administration compared with those of the C–C group. The C–HF group had higher BG at 30 min than HF–HF group (*P*<0.01). Furthermore, area under the curve (AUC) in C–HF and HF–HF groups was also significantly larger than C–C group (*P*<0.001). Fasting BG and homoeostasis model assessment of insulin resistance (HOMA-IR) of the offspring were significantly higher in C–HF and HF–HF groups than C–C group at 32 weeks of age (*P*<0.05). Operational taxonomic unit (OTU), Chao and Shannon indexes showed a significantly lower diversity in C–HF offspring compared with HF–C and C–C groups (*P*<0.05). The dominant phyla of all the groups were *Bacteroidetes*, *Firmicutes* and *Proteobacteria*, which also showed significantly different percentages in the group (*P*<0.05). Furthermore, it is indicated that *Lactobacillus* and *Bacteroides* were significantly associated with glucose response to a glucose load (*P*<0.05). In conclusion, maternal and post-weaning diet interaction predisposes the offspring to aberrant glucose metabolism and alterations of gut microbiota in later life. Our study is novel in focusing on the effects of maternal and post-weaning diet interaction on offspring gut microbiota and glucose metabolism in later life.

## Abstract (precis)

Our data suggest that maternal and post-weaning high-fat (HF) diet predisposes the offspring for obesity, glucose intolerance and altered gut microbiota in later life. Our work is novel in showing the modulation of gut microbiota between maternal diet and glucose homoeostasis.

## INTRODUCTION

The developmental origins of health and disease (DOHaD) hypothesis, firstly formulated in the early 1990s, proposes that imbalanced diet during early life plays a critical role in determining the risks of developing some metabolic diseases such as obesity, insulin resistance and diabetes mellitus in offspring [1]. Previous and recent studies have indicated that a maternal high-fat (HF) diet [2] and/or a postnatal weaning diet rich in fat [3] can increase susceptibility to obesity, glucose intolerance and insulin resistance in the adult offspring. However, the mechanisms underlying maternal imbalanced nutrition and such metabolic diseases in the offspring remain unclear.

Recently, gut microbiota, which can affect numerous biological functions throughout the body, has become a major and promising research area in biomedicine. The gut microbiota comprises 100 trillion bacteria generating a biomass of approximately 1–2 kg with 2000 distinct species, comprising a total genome of approximately 150 times as big as human genome [4]. Previous studies have suggested that gut microbiota is important in regulating metabolic pathways in healthy people and in patients with obesity, diabetes and cardiovascular diseases [5]. Numerous studies have demonstrated that the gut microbiota may play a key role in the development of obesity [6]. One study indicated that the gut microbiota derived from twins discordant for obesity could modulate metabolism in mice and it also revealed transmissible, rapid and modifiable effects of diet-by-microbiota interactions [7]. Another recent large human study provided further insights on the role of certain microbiotal members in obesity and insulin resistance [8]. It is indicated that obesity, insulin resistance and fatty liver were more prevalent in subjects of low bacterial richness [low gene count (LGC)], compared with those characterized by high gene count (HGC) [8]. The first metagenome-wide association study (MGWAS) showed that patients with Type 2 diabetes mellitus (T2DM) were characterized by a moderate degree of gut microbial dysbiosis, decreased butyrate-producing bacteria and increased various opportunistic pathogens, as well as an enrichment of other microbial whose functions are conferring sulfate reduction and oxidative stress resistance [9].

Most organs including liver, adipose tissue, pancreas, skeletal muscle and brain appear to be imprinted by these early disturbances [10]. However, little information is known about the gastrointestinal tract (GIT) in this programme and it is important given the central role of the GIT as a barrier between the outer environment such as diets and inner nutrient absorption and metabolism [11]. In the recent years, the gut microbiota is gradually known to be influenced by diet composition [12]. However, investigations into the later-life effects of these diets on gut microbiota in the offspring are limited. Our present objective was to explore the effect of maternal and post-weaning diet interaction on offspring's gut microbiota and glucose homoeostasis in later life.

## MATERIALS AND METHODS

### Ethics statement

All procedures were approved by the Animal Care and Use Committee of the Peking Union Medical College Hospital (Beijing, China, MC-07-6004) and were conducted in compliance with the Guide for the Care and Use of Laboratory Animals, 8th ed., 2011.

### Animal and diets

A total of ten male and 20 virgin female and male C57BL/6J mice (7 weeks old) that were specific pathogen free (SPF), were obtained from the Institute of Laboratory Animal Science, Chinese Academy of Medical Sciences and Peking Union Medical College (Beijing, China, SCXK-2013-0107). The animals were raised and kept under SPF conditions (room temperature at 22±2°C; 12 h light/dark cycle). Food and sterile water were provided ad libitum throughout the study period. Before mating, all the mice were randomly fed with the control (C) diet for 1 week for adaptation. Mating was performed by housing females with adult males fed with C diet for 3 days (male:female=1:2). Females were checked daily for postcopulatory plugs, and the presence of a plug in the morning after mating was taken as day 0.5 day of pregnancy.

Then, the pregnant mice were randomly assigned to two groups and were fed on either a C diet or C diet during pregnancy and lactation. The purified HF diet was composed of 58% of energy as fat, 25.5% carbohydrates and 16.4% protein. The purified C diet was composed of 11.4% of energy as fat, 62.8% carbohydrates and 25.8% protein. Both the C diet and HF diet were produced by Research Diets and the ingredient compositions were shown in Table 1. At day 1 after birth, the litter size of both the HF and C groups was standardized to six pups, to ensure no litter was nutritionally biased. All offspring were weaned at 3 weeks of age. At weaning, only one male offspring in each group was randomly selected from one litter. The male offspring of dams fed on either a HF diet or C diet and then weaned to either a HF or C diet for the following 29 weeks, generating four experimental groups: C–C (*n*=8), HF–C (*n*=8), C–HF (*n*=8) and HF–HF (*n*=8), which represents pregnancy and lactation compared with the post-weaning diet respectively. Male offspring were maintained until the end of the experimental period. The female offspring were not examined in our present study in order to prevent confounding factors related to their hormone profile and oestrus cycle.

**Table 1:** Nutritional composition of the C and HF diet fed to mice

	C diet		HF diet	
Ingredients	g	kcal	g	kcal
Casein	200	800	228	912
Amino acid	3	12	2	0
Corn starch	397	1590	0	0
Maltodextrin	132	528	170	680
Sucrose	100	400	175	700
Soya bean oil	70	630	25	225
Choline bitartrate	2.5	0	2	0
Mineral	35	0	40	0
Vitamin	10	40	10	40
Coconut oil	0	0	333.5	3001.5
**kcal/g**	**3.9**	**5.56**		

### Exsanguination and sampling

At the end of the experimental period (32 weeks of age), the male mice offspring were killed. Blood samples were collected from the intraorbital retrobulbar plexus after 10 h fasted anesthetized mice. Then, caecal and rectal content were quickly removed, snap frozen on top of dry ice, and stored at −80°C for further analysis, as previously described [13].

### Food intake and body weight

The offspring had free access to food. Seventy-two hours food intake was measured biweekly from 3 to 32 weeks old and their average food intake (g) was calculated by carefully collecting and weighing the remaining food including the spillage in the cage, and then subtracting the total remaining food from the known amount given before. The total energy (kJ) consumed by the offspring was calculated by multiplying the food intake (g) by the energy information (kJ/g) according to the nutritional composition of the HF and C diet fed to mice. Body weight was measured at birth, at weaning and per week post-weaning for each mouse.

### Glucose tolerance tests

After the mice had fasted for 10 h, glucose (2.0 g/kg body weight) was intraperitoneally administered. Blood glucose (BG) levels were measured before injection (time 0) and at 30, 60 and 120 min after injection from a tail bleed using a Contour TS glucometer (Bayer). The area under the curve (AUC) of intraperitoneal glucose tolerance test (IPGTT) was calculated by the trapezoid formula: AUC={0.5 × [(BG0 + BG30)/2]} + {0.5 × [(BG30 + BG60)/2]} + {1 × [(BG60 + BG120)/2]} [14].

### Biochemical analyses

At 32 weeks of age of the mice offspring, blood samples were collected after killing. The blood samples were centrifuged at 4000 ***g*** for 10 min and serum was stored in aliquots at −80°C. Serum insulin concentration was measured using the Mouse Ultrasensitive Insulin ELISA kit from ALPCO Diagnostics (80-INSMSU-E01). The intra-assay coefficients of variation for insulin measurements were 4.2%. Insulin sensitivity was assessed using the homoeostasis model assessment of insulin resistance (HOMA-IR). The HOMA-IR was calculated as fasting insulin concentration (μ units/ml) × [fasting glucose concentration (mmol/l)/22.5] [15].

### Microbial diversity analysis

#### DNA extraction and PCR amplification

Microbial diversity was analysed according to our recent publication [16]. Microbial DNA was extracted from caecal and rectal content samples using the E.Z.N.A.® Soil DNA Kit (Omega bio-tek), according to manufacturer's protocols. The V3–V4 region of the bacteria 16S rRNA gene were amplified by PCR (95°C for 2 min, followed by 27 cycles at 95°C for 30 s, 55°C for 30 s and 72°C for 45 s and a final extension at 72°C for 10 min) using primers 338F 5′-ACTCCTACGGGAGGCAGCA-3′ and 806R 5 ′-GGACTACHVGGGTWTCTAAT-3′, where barcode is an N-base sequence (N represent a unique 6–8 nt barcode) unique to each sample. PCR reactions were performed in 20 μl mixture containing 4 μl of 5× Fast Pfu Buffer, 2 μl of 2.5 mM dNTPs, 0.8 μl of each primer (5 μM), 0.4 μl of Fast Pfu Polymerase and 10 ng of template DNA. All reactions were carried out three biological replicates and each analysis consisted of three technical replicates.

#### Illumina MiSeq sequencing

Amplicons were extracted from 2% agarose gels and purified using the AxyPrep DNA Gel Extraction Kit (Axygen Biosciences) according to the manufacturer's instructions and quantified using QuantiFluor™-ST (Promega). Purified amplicons were pooled in equimolar and paired-end sequenced (2×300) on an Illumina MiSeq platform according to the standard protocols.

#### Bioinformatic analysis

Previous studies suggested that comparison of communities should be made using equal number of sequence reads in order to minimize the sequencing artefact as the number of spurious phylotypes increases with sequencing effort [17]. Therefore, we picked 14660 sequencing reads from each sample using a pseudorandom generator for comparison of community composition and structure among samples, due to that the lowest sequencing read of all the samples was 14660. Raw fastq files were demultiplexed, quality-filtered using QIIME (version 1.17) with the following three criteria: (1) The 300 bp reads were truncated at any site receiving an average quality score <20 over a 10 bp sliding window, discarding the truncated reads that were shorter than 50 bp. (2) Exact barcode matching, two nucleotides mismatch in primer matching, reads containing ambiguous characters were removed. (3) Only sequences that overlap longer than 10 bp were assembled according to their overlap sequence. Reads which could not be assembled were discarded. Operational taxonomic units (OTUs) were clustered with 97% similarity cut-off using UPARSE (version 7.1 http://drive5.com/uparse/) and chimaeric sequences were identified and removed using UCHIME. The phylogenetic affiliation of each 16S rRNA gene sequence was analysed by RDP Classifier (http://rdp.cme.msu.edu/) against the silva (SSU115) 16S rRNA database using confidence threshold of 70% [18].

### Statistical analysis

Statistical differences of body weight and glucose metabolism parameters were determined using ANOVA followed by multiple-comparison testing using Bonferroni post hoc analysis. The Mann–Whitney test was used to evaluate the relative abundance (%) of gut microbiota between indicated groups. Correlation analyses between relative abundance of sequences belonging to different bacterial class and glucose response to a glucose load were performed by using Spearman's correlation analyses. *P* value <0.05 were considered to indicate statistical significance. All statistical analyses were calculated with SPSS 21.0 (SPSS).

## RESULTS

### Body weight and food intake

**Figure 1:**
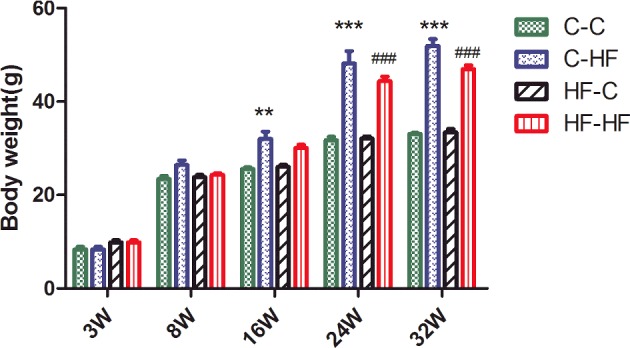
Body weight for male offspring from 3 to 32 weeks Data represent as mean ± S.D. (*n*=8/in each group). Statistical differences were determined using ANOVA followed by multiple-comparison testing using Bonferroni post hoc analysis; **P<0.05 C–HF compared with the C–C group; ***P<0.001 C–HF compared with the C–C group; ^###^*P*<0.001 HF–HF compared with the C–C group. Dam and pup diets denoted before and after the dash line respectively.

Body weight at birth and weaning were similar among the different diet groups. The offspring in C–C group had lower body weight throughout the study compared with C–HF and HF–HF groups. Specifically, the C–C offspring had body weight than C–HF group at 16 weeks of age (*P*<0.01) and both C–HF and HF–HF offspring had higher body weight than C–C group at 24 and 32 weeks of age (*P*<0.001 respectively). However, there is no significant difference between other groups (Figure 1). No difference in food and water consumption was observed among the groups throughout the experiment (results not shown).

### Glucose response

**Figure 2:**
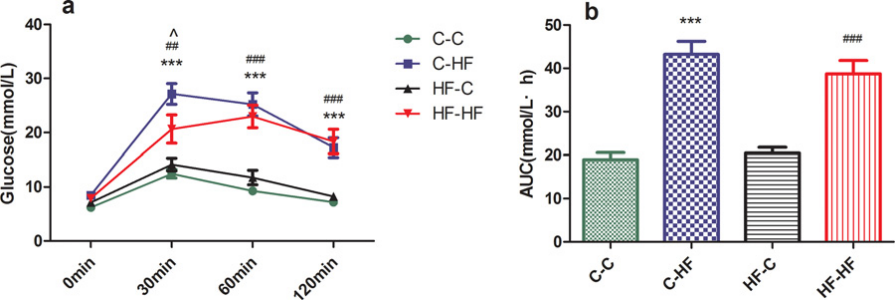
IPGTT (**a**) and AUC (**b**) for male offspring at 32 weeks Data represent as mean ± S.D.(*n*=8/each group). Statistical differences were determined using ANOVA followed by multiple-comparison testing using Bonferroni post hoc analysis; ***P<0.001 C–HF compared with the C–C group; ^##^*P*<0.01, ^###^*P*<0.001 HF–HF compared with the C–C group; ^∧^*P*<0.05 HF–HF compared with the C–HF group. Dam and pup diets denoted before and after the dash line respectively.

The BG levels of the male offspring from the C and HF dams weaned HF diet were significantly higher at 30 min (*P*<0.001 and *P*<0.01 respectively), 60 min (*P*<0.001) and 120 min (*P*<0.001) after intraperitoneal glucose administration compared with those of the C–C group. The C–HF group had higher BG at 30 min than HF–HF group (*P*<0.01) (Figure 2a). Furthermore, AUC in C–HF and HF–HF groups was also significantly larger than C–C group (*P*<0.001). However, no difference in BG and AUC was observed between HF–C and C–C groups throughout the experiment (Figure 2b).

### Insulin resistance

**Figure 3:**
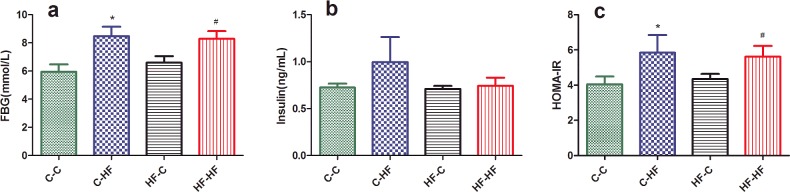
Fasting BG (**a**), serum insulin levels (**b**) and HOMA-IR (**c**) for male offspring at 32 weeks Data represent as mean ± S.D.(*n*=8/each group). Statistical differences were determined using ANOVA followed by multiple-comparison testing using Bonferroni post hoc analysis; *P<0.05 C–HF compared with the C–C group; ^#^*P*<0.05 HF–HF compared with the C–C group. Dam and pup diets denoted before and after the dash line respectively.

To determine insulin sensitivity of the offspring, the levels of serum fasting BG and insulin were determined. There was no significant difference between the four groups (*P*<0.05, Figure 3b). However, fasting BG and HOMA-IR of the offspring were significantly higher in C–HF and HF–HF groups than C–C group at 32 weeks of age (*P*<0.05). However, there is no significant difference between HF–C and C–C groups (Figures 3a and 3c).

### Characteristics of sequencing results

The offspring in all the four groups (*n*=6, each group) of microbial diversity were investigated. A total of 351840 high-quality sequences were produced in the present study, with an average of 14660 sequences per sample (Table 2). The Good's coverage of each group was over 97%, indicating that the 16S rDNA sequences identified in these groups represent the majority of bacteria present in the samples of the present study. The OTU, the estimators of community richness (Chao) and diversity (Shannon) are also shown in Table 2. Lower OUT, Chao and Shannon indexes were observed in C–HF group and lower Chao was found in HF–HF group, compared with HF–C and C–C groups, demonstrating the significantly lower richness and diversity found in C–HF offspring compared with HF–C and C–C groups. And there were no significant differences of OUT, Chao and Shannon indexes between HF–C and C–C groups. Group HF–HF had a lower median OUT and Shannon indexes compared with the C group, but the difference was not significant (Table 2).

**Table 2:** Sequencing data summary Data represent as mean ± S.D. (*n*=6, in each group). Statistical differences were determined using ANOVA followed by multiple-comparison testing using Bonferroni post hoc analysis. The number of OTUs, richness estimator Chao and diversity estimator Shannon were calculated at 3% distance. *P<0.05 C–HF compared with C–C group, ^∧^*P*<0.05 C–HF compared with HF–C group, ^#^*P*<0.05 HF–HF compared with C–C group, ^$^*P*<0.05 HF–HF compared with HF–C group. Dam and pup diets denoted before and after the dash line respectively.

	C–C	HF–C	C–HF	HF–HF
Sequences	14660.00	14660.00	14660.00	14660.00
OTUs	239.3±6.1	262.7±9.6	108.7±68.4*^∧^	195.0±29.5
Chao	265.0±14.8	289.3±7.1	129.3±63.6*^∧^	220.7±19.7^#$^
Shannon	3.8±0.1	4.2±0.2	2.7±0.8*^∧^	3.3±1.2

### Principal coordinates analysis among different groups

**Figure 4:**
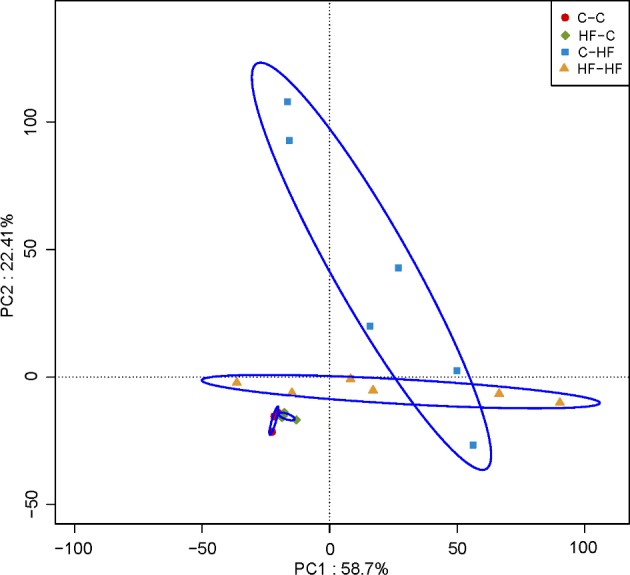
PCA plots of gut communities in the offspring *n*=6/each group; dam and pup diets denoted before and after the dash line respectively.

Closer analysis of bacterial differences induced by diet was determined by sequencing the 16S rRNA encoding genes present in the caecal and rectal content. Principal coordinates analysis (PCA) of Illumina MiSeq amplicon data demonstrated significantly separate clustering of the gut communities in the offspring among the four groups with PC1 percent variation explained=58.7% and PC2 percent variation explained=22.41%. It is indicated that there were significant difference of microbial composition offspring between post-weaning HF diet and C diet. However, no marked differences in the microbial composition were observed between HF–C and C–C groups (Figure 4).

### Microbial structures of the offspring differed significantly

**Figure 5:**
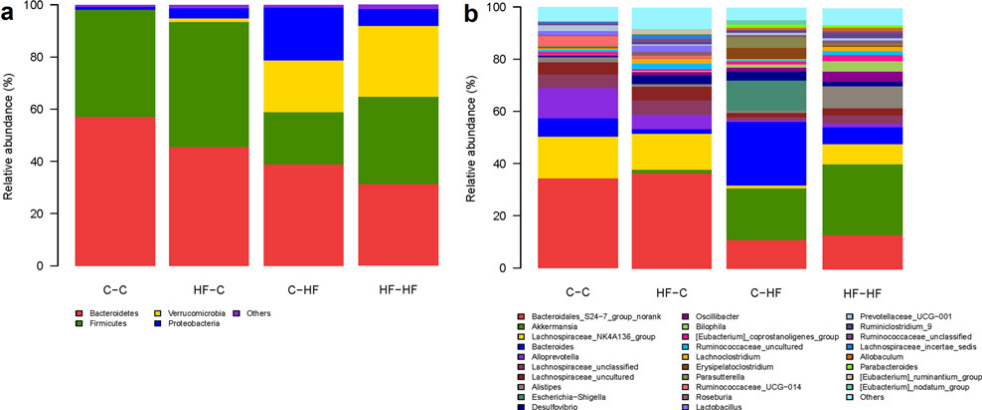
Relative abundance of bacterial phyla in microbiota in each group at the phylum (**a**) and genus (**b**) level *n*=6/each group; dam and pup diets denoted before and after the dash line respectively.

**Figure 6:**
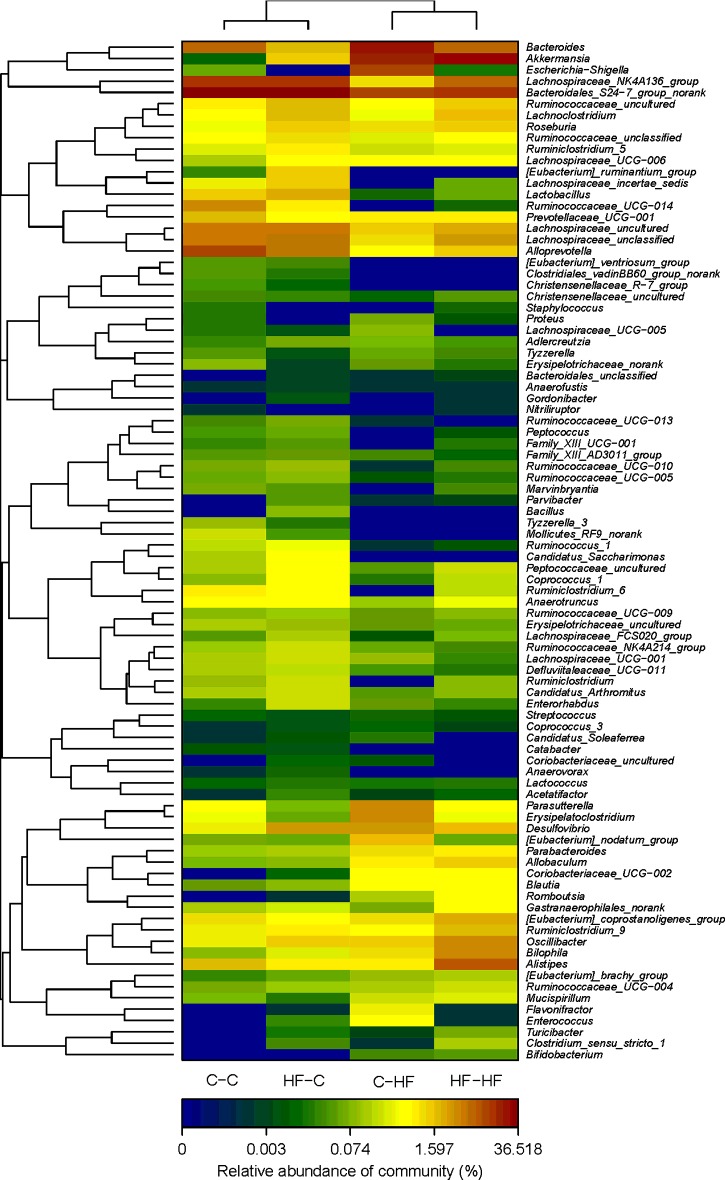
Heat map analyses of abundant genera in each group *n*=6/each group; the *y*-axis is a neighbour-joining phylogenetic tree, each row is a different phylotype. The abundance plot shows the proportion of 16S rRNA gene sequences in each group. Dam and pup diets denoted before and after the dash line respectively.

The overall microbiota structure for each group at the phylum and genus level is shown in Figure 5. The dominant phyla of all the four groups were *Bacteroidetes*, *Firmicutes* and *Proteobacteria*. The *Verrucomicrobias* were significantly increased in C–HF and HF–HF groups, compared with C–C and HF–C groups and *Bacteroidetes* were significantly decreased in both C–HF and HF–HF groups. At the phylum and genus level, the relative abundance of microbiota sequences both revealed that C–HF microbiota exhibited a closer similarity to HF–HF group, and HF–C microbiota exhibited a closer similarity to C–C offspring. The heat map according to bacterial genus level also demonstrated the same phenomenon that the microbial structures of HF–C and C–C groups can be clustered together (Figure 6).

### Phylotypes significantly different in offspring mice

There were significant variations in the composition of the caecal and rectal content in the four groups at different bacterial levels. At the phylum level, *Bacteroidetes, Firmicutes* and *Saccharibacteria* were significantly decreased in C–HF group, compared with C–C group (*P*<0.05). *Bacteroidetes* and *Saccharibacteri*a were significantly decreased, whereas *Proteobacteria* was significantly decreased in HF–HF offspring, compared with C–C offspring. At the genus level, with ten significantly different genera between C–HF and C–C groups, and five different genera between HF–HF and C–C groups. Specifically, total eight genera of *Bacteroidales_S24-7_group_norank, Candidatus_Saccharimonas, Defluviitaleaceae_UCG-011, Lachnospiraceae_NK4A136_group, Lachnospiraceae_uncultured, Lactobacillus, Marvinbryantia* and *Ruminococcus_1* showed a significant lower percentage in C–HF group (*P*<0.05), whereas *Bacteroides and Erysipelatoclostridium* were increased in C–HF group, compared with C–C group (*P*<0.05). All five genera of *Bacteroidales_S24-7_group_norank, Candidatus_Saccharimonas, Defluviitaleaceae_UCG-011, Lac-tobacillus* and *Ruminococcus_1* exhibited a statistically significant lower percentage in HF–HF group than C–C group. Furthermore, in order to determine the effects on microbial composition in the offspring exposed to different maternal diets during pregnancy and lactation, we compared the microbial composition between HF–C and C–C groups. It is indicated that there was no significant differences between HF–C and C–C groups at the phylum level. And only one genus of *Bacteroides* was lower in HF–C group, compared with C–C group (Table 3).

**Table 3:** Phylotypes significantly different in offspring groups Statistical analysis was performed by Mann–Whitney test. Data of C–C, C–HF and HF–HF groups were relative abundance (percentage) of all sequences in each group. *P<0.05 C–HF compared with the C–C group, ^#^*P*<0.05 HF–HF compared with the C–C group, ^&^*P*<0.05 C–HF compared with the C–C group; n.s.: no significance. Dam and pup diets denoted before and after the dash line respectively.

Taxonomic rank		C–C (%)	HF–C (%)	*P* value	C–HF (%)	HF–HF (%)	*P* value	*P* value
Phylum	*Actinobacteria*	0.05	0.38	0.09	0.67	0.75	0.14	0.35
Phylum	*Bacteroidetes*	57.10	45.59	0.22	38.85	31.18	0.04*	**0.02^#^**
Phylum	*Cyanobacteria*	0.13	0.28	0.49	0.07	0.68	0.49	0.26
Phylum	*Deferribacteres*	0.08	0.02	0.09	0.24	0.31	0.37	0.41
Phylum	*Firmicutes*	41.09	47.82	0.48	20.00	33.46	0.04*	0.65
Phylum	*Proteobacteria*	1.10	3.87	0.12	20.28	6.43	0.19	**0.04^#^**
Phylum	*Saccharibacteria*	0.17	0.64	0.08	0.00	0.00	0.02*	**0.02^#^**
Phylum	*Verrucomicrobia*	0.01	1.37	0.39	19.89	27.19	0.16	0.25
Genus	*Alloprevotella*	11.50	5.49	0.31	0.70	1.47	0.05	0.07
Genus	*Bacteroidales_S24-7_group_norank*	34.41	36.52	0.56	10.96	13.14	0.00*	**0.00^#^**
Genus	*Bacteroides*	7.05	1.84	**0.02^&^**	24.30	6.44	0.03*	0.75
Genus	*Candidatus_Saccharimonas*	0.17	0.64	0.08	0.00	0.00	0.02*	**0.02^#^**
Genus	*Christensenellaceae_uncultured*	0.03	0.02	0.49	0.01	0.04	0.07	0.72
Genus	*Clostridiales_vadinBB60_group_norank*	0.04	0.02	0.35	0.00	0.00	0.06	0.06
Genus	*Defluviitaleaceae_UCG-011*	0.15	0.20	0.38	0.03	0.02	0.03*	**0.01^#^**
Genus	*Erysipelatoclostridium*	0.46	0.06	0.12	4.27	0.46	0.04*	1.00
Genus	*Lachnospiraceae_NK4A136_group*	15.93	13.85	0.58	1.07	7.68	0.00*	0.14
Genus	*Lachnospiraceae_unclassified*	5.21	5.48	0.86	1.17	3.14	0.07	0.40
Genus	*Lachnospiraceae_uncultured*	4.78	5.21	0.52	1.58	2.73	0.02*	0.15
Genus	*Lactobacillus*	1.53	2.30	0.57	0.01	0.06	0.00*	**0.00^#^**
Genus	*Marvinbryantia*	0.06	0.05	0.38	0.00	0.03	0.03*	0.35
Genus	*Parabacteroides*	0.13	0.13	0.96	1.16	0.93	0.07	0.09
Genus	*Peptococcaceae_uncultured*	0.17	0.48	0.10	0.04	0.23	0.05	0.22
Genus	*Ruminiclostridium_6*	0.97	0.56	0.42	0.00	0.18	0.09	0.17
Genus	*Ruminococcus_1*	0.21	0.46	0.36	0.00	0.01	0.03*	**0.03^#^**

### Correlation analyses

**Figure 7:**
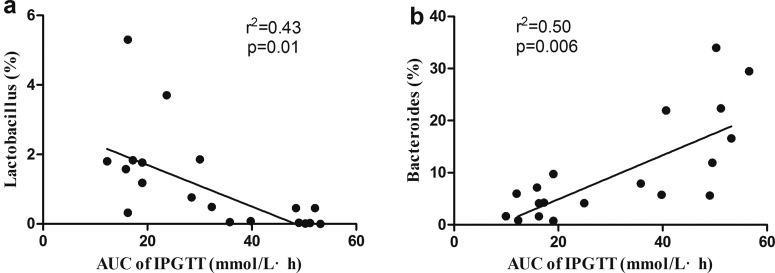
Correlation analyses between relative abundance (%) of sequences belonging to Lactobacillus (a), Bacteroides (b) genus and glucose response to a glucose load *n*=6/each group.

Correlation analyses between relative abundance (percentage) of sequences belonging to a specific bacterial genus and glucose response to a glucose load were performed by using Spearman's correlation analyses. The glucose response to a glucose load was assessed by AUC of IPGTT. Interestingly, it is indicated that *Lactobacillus* percentage were negatively associated with AUC of IPGTT (*r*^2^=−0.43, *P*=0.01) and *Bacteroides* percentage were positively associated with AUC of IPGTT (*r*^2^=0.5, *P*=0.0006) (Figure 7).

## DISCUSSION

Our study was designed to examine the effect of maternal and post-weaning HF diet interaction on offspring's gut microbiota and glucose homoeostasis in later life. This long-term HF diet mice model is particularly important in the light of the increasing consumption of refined foods with a high glycaemic index and fat content among men and women now consuming 30% more saturated fats than the recommended daily intake [19]. Similar to other studies [20–22], we have shown that offspring from both HF and C dams fed on HF diet from weaning to adulthood had heavier body weight, severer impaired glucose tolerance and lower insulin sensitivity level at 32 weeks of age. It indicated that maternal and post-weaning HF diet interaction could contribute to abnormal glucose metabolism in the later life of offspring.

Our present study indicated that both HF–HF and C–HF offspring had lower bacterial richness. Similarly, one large human study also demonstrated that individuals with a low bacterial richness are characterized by more marked overall adiposity, insulin resistance and dyslipidaemia, which indicated that a low bacterial richness may be associated with abnormal glucose metabolism [8]. The dominant phyla of all the groups were *Bacteroidetes*, *Firmicutes* and *Proteobacteria*. These data were consistent with findings from Karlsson et al. [13] that assessed the long-term effects of a high-energy-dense diet, supplemented with *Lactobacillus plantarum (Lp)* or *Escherichia coli (Ec)* on weight gain, fattening and the gut microbiota in rats. More specifically, *Tenericutes*, *Bacteroidete*, *Lactobacillus*, *Ruminococcus* and *Prevotella* significantly decreased whereas *Proteobacteria* significantly increased in both C–HF and HF–HF groups. Jumpertz et al. [23] indicated that a corresponding decrease in *Bacteroidetes* was associated with an increased energy harvest of approximately 150 kcal/day in one inpatient study. Similar findings were found in a human study which showed the relative abundance of *Bacteroides* in the T2DM group (approximately 10%) was lower than that in the normal glucose tolerance and prediabetes groups [24]. Another study indicated that large alterations were associated with switching to the HF diet of wild-type mice, including a decrease in *Bacteroidetes* and an increase in *Proteobacteria*. These changes were seen for both genotypes (i.e. in the presence and absence of obesity), which indicated that it was the HF diet itself, and not the obese state, mainly accounting for the observed alterations in the gut microbiota. These findings demonstrated the importance of diet as a determinant of gut microbiome composition and suggest the need to C dietary variation when evaluating the composition of the human gut microbiome [25].

Of interest, our present study showed *Lactobacillus* percentage was negatively associated with glucose response to a glucose load and a corresponding positive correlation between *Bacteroides* and glucose response to a glucose load. It indicated gut microbiota such as *Lactobacillus* and *Bacteroides* might be important bacterial phyla in regulating glucose metabolism in the offspring and they can be the target of diabetes prevention and intervention. Recently, numerous studies have indicated that *Lactic acid bacteria (LAB)* are useful in preventing or delaying the onset of diabetes [26]. One study found that the BG levels were significantly lower in Type 2 diabetic mice which were administered *Lactobacillus gasseri BNR17* than those in a C group [27]. The underlying mechanism may be that *LAB* can enhance pancreatic glutathione biosynthesis, thus reducing oxidative stress in the pancreas [28].

In conclusion, our data suggest that maternal and post-weaning HF diet interaction predisposes the offspring for obesity, glucose intolerance, insulin resistance and alters gut microbiota in later life. Our work is novel in showing the modulation of gut microbiota between maternal and post-weaning diet interaction and glucose homoeostasis. Therefore, as both ‘gut microbiota’ and ‘fetal programming’ become more evident and prevalent in obesity and T2DM, a better understanding of the role of the gut microbiota in ‘fetal programming’ might provide novel perspectives regarding its pathophysiological relevance and pave the way for new therapeutic principles of obesity and T2DM.

## Abbreviations

AUC: area under the curve
BG: blood glucose
C: control
GIT: gastrointestinal tract
HF: high-fat
HOMA-IR: homoeostasis model assessment of insulin resistance
IPGTT: intraperitoneal glucose tolerance test
*LAB*: *Lactic acid bacteria*
OTU: operational taxonomic unit
PCA: principal coordinates analysis
SPF: specific pathogen free
T2DM: Type 2 diabetes mellitus
